# Supplementary material for: Fragmentation of production amplifies systemic risks from extreme events in supply-chain networks
Source: PLoS One. 2020 Dec 28;15(12):e0244196. doi: 10.1371/journal.pone.0244196 (PMC7769560; doi:10.1371/journal.pone.0244196)
Supplement: S1 File — (DOCX) [file pone.0244196.s001.docx]

Supplementary information is composed of three parts.

1. Sections S1 to S5 present analytical results for simple supply chains. They allow us to identify the mechanisms that drive the results shown in the Main Text. Section S1 to S4 focus on a single producer connected to multiple primary producers. The dynamical equations for inventory and profit are established in Section S1. Section S2 shows how the overordering rate that maximizes profits changes with parameters. Section S3 describes the mechanism that determines this behavior for fully durable goods. Section S4 presents the analytical treatment for fully perishable goods. Section S5 shows a method to algorithmically compute the exact expected values of profits and overordering for a large class of complex supply chain.
2. Sections S6 and S7 provide information on the evolutionary process. Section S6 describes in detail the evolutionary process used in the simulations. Section S7 provides evidence on the existence of evolutionary equilibria.
3. Sections S8 to S10 present additional numerical results. Section S8 shows a topologically periodic distribution of the overordering rates obtained with a layered supply chain. Section S9 presents the distribution of the mitigation success as a function of fragmentation. Section S10 presents the statistical correlations between the profit-maximizing overordering rates and a selection of ten network indicators.


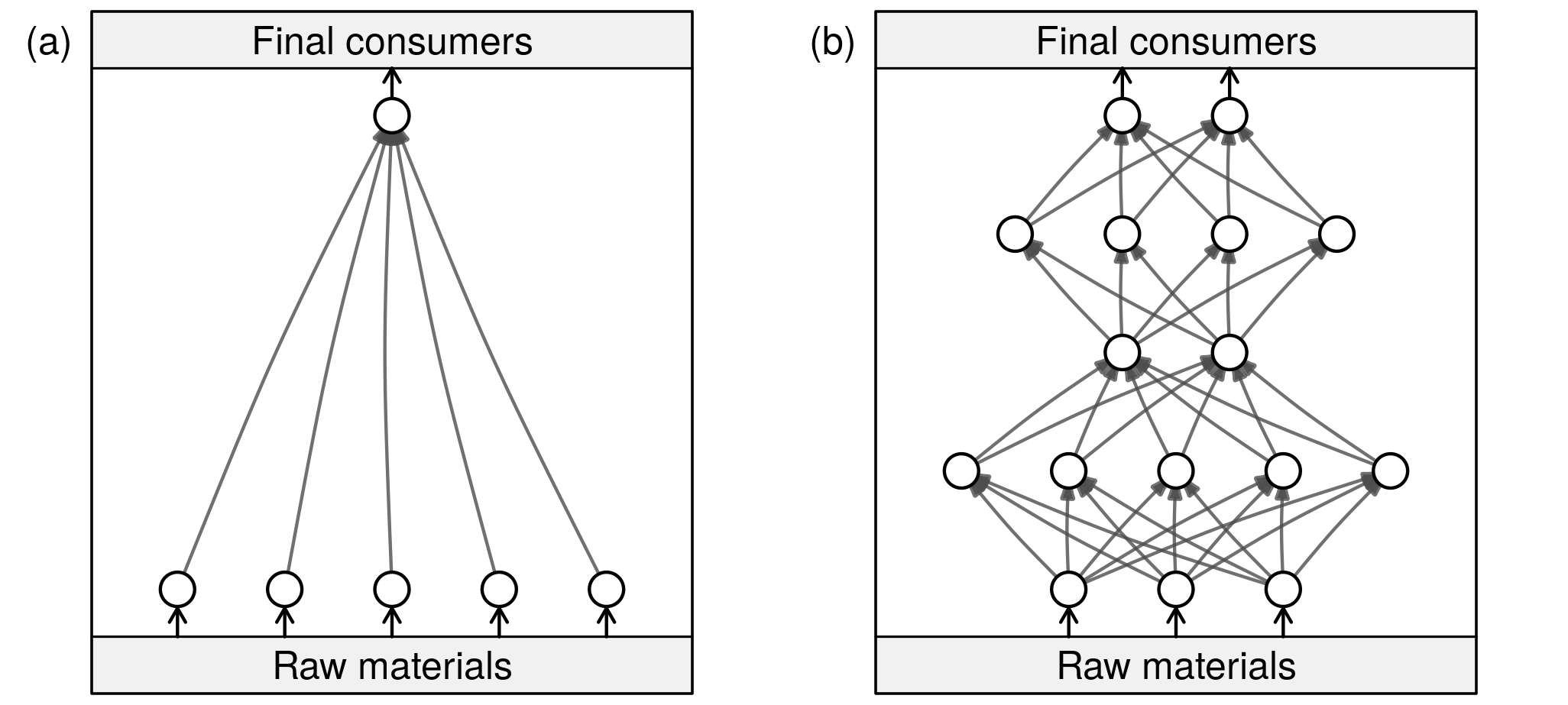


**Fig. S1.** Analytical results are derived for simpler supply chains. Panel (a) displays a two-layer supply chain with one final producer supplied by five primary producers. Panel (b) shows a fully connected five-layer supply chain. The network topology is summarized by the vector $(3,5,2,4,2)$, which gives the number of firms that corresponds to each one of the five layers: 3 primary producers, 5 layer-two firms, …, and 2 final producers. This supply chain is fully connected because each of the layer-$l$ firms is supplied by all of the layer-$(l-1)$ firms, for $l=2, \ldots, 5$.

## S1. A single producer supplied by multiple firms: Dynamical equations

This situation is illustrated in Fig. S1(a) for $s=5$ suppliers. The $s$ suppliers are primary producers. Since the inflow of raw materials is not subject to external perturbations, they do not overorder. We focus on the inventory of the final producer, denoted by $h_{t}$. It changes through time as follows:

|  | $h_{t+1}=h_{t}+\mathcal{I}_{t}-\mathcal{O}_{t}-\left( 1-v \right)\left( r_{t}+\mathcal{I}_{t}-\mathcal{O}_{t} \right),$ | [A1] |
| --- | --- | --- |

where $\mathcal{I}_{t}$ is the quantity received, $\mathcal{O}_{t}$ the quantity used for production. The last term represents the impact of non-durability: a share $1-v$ of the remaining inventory is lost at each time step. This expression can be rewritten as follows:

|  | $h_{t+1}=v\left( r_{t}+\mathcal{I}_{t}-\mathcal{O}_{t} \right).$ | [A2] |
| --- | --- | --- |

We denote by $K_{t}$ the random variable representing the number of externally perturbed suppliers at time $t$. This variable — which can take up to $s+1$ values: $0, 1, \ldots, s$ — follows a binomial distribution:

|  | $\Pr\left( K=k \right)=\left( \begin{matrix} s \\ k \end{matrix} \right)p^{k}\left( 1-p \right)^{s-k}.$ | [A3] |
| --- | --- | --- |

When $K_{t}$ suppliers are perturbed, the quantities $\mathcal{I}_{t}$ and $\mathcal{O}_{t}$ are:

|  | $\mathcal{I}_{t}=\frac{s-K_{t}}{s}\frac{1+r}{z},$ | [A4] |
| --- | --- | --- |
|  | $\mathcal{O}_{t}=\min\left( \frac{1}{z},r_{t}+\mathcal{I}_{t} \right).$ | [A5] |

To eliminate the $\min$function, we determine the maximum number of suppliers that can simultaneously fail without impacting final production. It is the largest $K_{t}$ such that $h_{t}+\mathcal{I}_{t}\geq1/z$. Using Eq. [S4] in the inequality, this number is the largest integer lower than or equal to:

|  | $\bar{s}_{t}=\frac{s r+z h_{t}}{1+r}.$ | [A6] |
| --- | --- | --- |

We can thus rewrite Eq. [S2] by distinguishing whether is threshold is crossed:

|  | $h_{t+1}=\left\{ \begin{aligned} v\left( h_{t}+\frac{r}{z}-\frac{K_{t}}{s}\frac{1+r}{z} \right), \mathrm{if}K_{t}\leq\frac{sr+zh_{t}}{1+r}, \\ 0, \mathrm{otherwise}. \end{aligned} \right.$ | [A7] |
| --- | --- | --- |

The profit — sales minus input costs — can be derived from the inventory:

|  | $\pi_{t}=\left\{ \begin{aligned} P_{t}-\frac{s-K_{t}}{s}\frac{1+r}{z}, \mathrm{if}K_{t}\leq\frac{sr+zh_{t}}{1+r}, \\ P_{t}zh_{t}+\frac{s-K_{t}}{s}\left( 1+r \right)\left( 1-p- \frac{1}{z} \right), \mathrm{otherwise}, \end{aligned} \right.$ | [A8] |
| --- | --- | --- |

where $P_{t}$ is a random variable equals to 0 with probability $p$ and 1 otherwise. If either inventory or overordering gets larger, the threshold value $\bar{s}_{t}$ also grows. The firm is less vulnerable to supply disruption and meets the full demand more often; see the first Eq. of [S8]. If both inventory and overordering are low enough, a combination of perturbations may induce a shortage. The entire inventory is used for production and a share of the demand remains unmet; see the second equations of [S5] and [S6].

## S2. A single producer supplied by multiple firms: A summary of the influence of parameters on the optimal overordering rate

We present how the overordering rate that maximizes the expected profit of the final producer, denoted by$r^{*}$, changes with durability$v$, failure rate $p$, productivity $z$, and number of suppliers $s$. We focus on the productive region, defined by $p<1-1/z$. Results are shown in Fig. S2, and are commented on for three scenarios of durability.

- When $v=100\%$, there is a smooth continuous relationship between $r^{*}$ and $p$, namely: $r^{*}=p/\left( 1-p \right)$. This equation is not affected by $z$ or $s$. The mechanism underpinning this result is explained in Section S3.
- When $v=0\%$, the relationships between $r^{*}$ and $p$ and between $r^{*}$ and $z$ are discontinuous. The optimal rate $r^{*}$ can take up to $s$ values, equal to $i/\left( s-i \right)$ with $i=0, 1,\ldots,s-1$. It jumps from one of this value to another as $p$ or $z$ crosses specific threshold values; these values are explicitly derived in Section S4. With more numerous suppliers, a larger set of values is accessible to $r^{*}$, so that the firm can more finely manage a particular failure rate. For instance, considering the three $0\%$-durability curves in Fig. S2, we observe that it is never profitable to overorder with one or two suppliers, whereas for five suppliers the optimal rate $r^{*}$ jumps from 0 to 25% when $p$ crosses 25%.
- The behavior of $r^{*}$ for intermediate durability interpolates between the features described for $v=100\%$ and $v=0\%$. In Fig. S2, as $v$ decreases, a threshold behavior appears for low and high failure rates. Overordering is profitable only for intermediate failure rates. This $p$-interval gets reduced as $v$ decreases. The set of accessible $r^{*}$-values also gets smaller, leading to a more discontinuous behavior. Having more suppliers smooths these nonlinearities; see the $90\%$-durability curves across all three panels of Fig. S2.


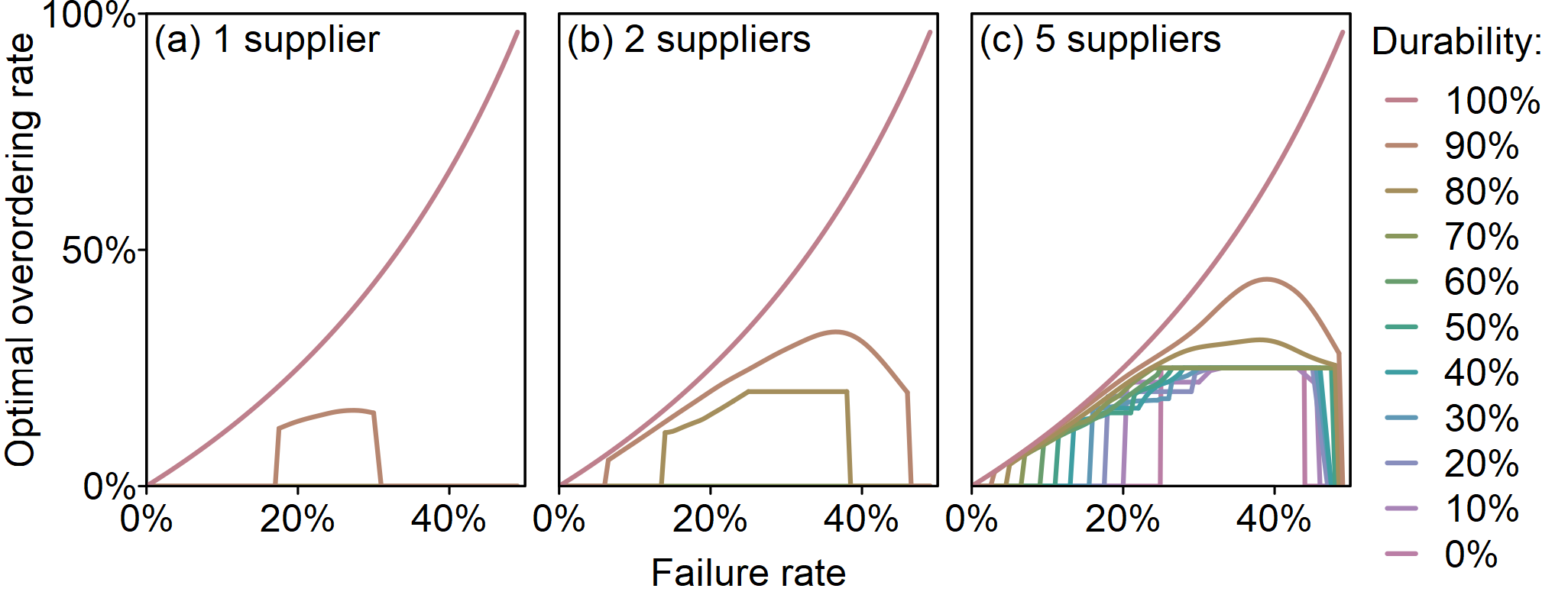


**Fig. S2.** Profit-maximizing overordering rates depends nonlinearly on parameters. These curves concern a final producer supplied by (a) one, (b) two, and (c) five suppliers, as illustrated in Fig. S1(a). The panels contain 11 curves, corresponding to different durabilities. Productivity is 2, and only the productive region of the parameter space is shown, (i.e., with failure rates between 0 to 50%).

## S3. Origin of the large optimal overordering rates when goods are fully durable

When the final producer is supplied by a single primary producer, the dynamics of its inventory, denoted by $x_{t}$, can be interpreted as a one-dimensional asymmetric random walk in the positive real line. At each time step, inventory increases by $r/z$ with probability $1-p$ or decreases by $\min\left( x_{t}, 1/z \right)$ with probability $p$. The origin of the positive real line, 0, is a so-called sticky wall: because of the $\min$function, any trajectory below $1/z$ that undergoes a negative step simply lands on the wall. This behavior is to be distinguished from that of reflecting walls, on which trajectories bounce back, and of absorbing walls, in which trajectories get caught. In this class of random walk, trajectories are expected to diverge if the expected growth rate ${\mathbb{E}\left( x_{t+1} \right)}/{x_{t}}$ is higher than one (i.e., when the positive step has a larger expected size than the negative step). Otherwise, trajectories are expected to sometimes touch the wall. In our model, this condition is $\left( 1-p \right)r/z\geq p/z$. The overordering rate that maximize profit, $r^{*}$, is the one that turns this inequality into an equality:

|  | $r^{*}=\frac{p}{1-p}.$ | [A9] |
| --- | --- | --- |

This solution can be explained by the following decision-making heuristics. When $r$ is higher than $p/{(1-p)}$, inventory eventually diverges. Consequently, most extra inputs purchased are not used, implying that the amount of sales could be achieved with less overordering. Conversely, when $r$ is lower than $p/{(1-p)}$, inventory is sometimes below $1/z$, which leaves the firm vulnerable to supply disruption, and some sales are missed. With fully durable goods, however, it is always profitable not to miss sales. When one unit of output is sold, cost only consists of the associated quantity of input consumed—there is no need to purchase extra inputs to replace an obsolete inventory. As a consequence, if sales are missed, it is profitable to increase overordering. This heuristics also applied when the firm has more than one supplier.

## S4. Nonlinear behavior of the optimal overordering rate when goods are not durable

We first aim to write an explicit equation of the expected profit of a final producer supplied by $s$ primary producers, as in Fig. S1(a), for $v=0\%$. To that end, we determine for one time step all the possible value of profit and their associated probability. Since inputs are not storable, the firm can only tap into the inputs received during the same time step. Costs are directly proportional to the number of failed suppliers, denoted by $k=0, \ldots, s$. Sales depend nonlinearly on $k$: they are proportional to $k$ but cannot exceed the final demand, which equals one. Combining Bernoulli distributions, we obtain the expression of the expected profit $\mathbb{E}\left( \pi\right)$:

|  | $\mathbb{E}\left( \pi\right)=\sum_{k=0}^{s} \left( \begin{matrix} s \\ k \end{matrix} \right)p^{k}\left( 1-p \right)^{s-k}\left[ \left( 1-p \right)\min\left( \frac{s-k}{s}\left( 1+r \right),1 \right)-\frac{s-k}{s}\frac{1+r}{z} \right].$ | [A10] |
| --- | --- | --- |

We note that the overordering rates of the primary producers do not affect $\mathbb{E}\left( \pi\right)$. The min function corresponds to the fact that sales cannot exceed the demand. To eliminate this function in [S8], we identify the largest $k$-value for which the $\min$ expression yields one. It is immediate that this value, denoted by $\hat{s}\left( r \right)$, is:

|  | $\hat{s}\left( r \right)=U\left( \frac{sr}{1+r} \right),$ | [A11] |
| --- | --- | --- |

where operator $U$ transforms a real number into the highest integer less than or equal to this number. Integer $\hat{s}\left( r \right)$ represents the maximum number of suppliers that, for a given level of overordering, can be shocked without loss of sales. Using $\hat{s}\left( r \right),$expression [S8] can be rewritten as:

|  | $\mathbb{E}\left( \pi\right)=(1-p)\left[ \sum_{k=0}^{\hat{s}\left( r \right)} \left( \begin{matrix} s \\ k \end{matrix} \right)p^{k}\left( 1-p \right)^{s-k}\left[ 1-\left( 1-\frac{k}{s} \right)\left( 1+r \right) \right]+\left( 1+r \right)\left( 1-p-\frac{1}{z} \right) \right].$ | [A12] |
| --- | --- | --- |

In the full fragmentation scenario, the objective of the final producer is to select the overordering rate $r^{*}$ that maximizes its expected profit $\mathbb{E}\left( \pi\right)$. We define on $\mathbb{R}^{+}$ the function $\mathcal{G:}r\mathbb{⟼E}\left( \pi\left( r \right) \right)$; it represents the fitness landscape. For any $z>1$, any $p\in\left[ 0,1 \right]$ and any $s\in\mathbb{N}^{*}$, function $\mathcal{G}$ is continuous and piecewise linear. As $r$ increases from $0\%$ onward, $\hat{s}\left( r \right)$ jumps from one integer value to the next, from $0$ all the way to $s-1$. Each time $\hat{s}\left( r \right)$ increases, a new term in the sum of [S9] is added, leading to a new linear piece for $\mathcal{G}$. The number of linear pieces of $\mathcal{G}$ is equal to the number of values that $\hat{s}\left( r \right)$ can take, which is $s$. Using Eq. [S11], we find the $(s-1)$ values of $r$ separating the $s$ pieces:

|  | $r^{i}=\frac{i}{s-i} ,$ | [A13] |
| --- | --- | --- |

with $i=1, \ldots, s-1$. We add to this ensemble the term $r^{0}=0$, which corresponds to the left boundary of the first linear piece. The last linear piece of $\mathcal{G}$ lies on the interval $\left[ r^{s-1},+\infty\right[$. There, given [S12], $\mathcal{G}$ is decreasing in $r$ with coefficient of variation $-{\left( 1-p \right)^{2}}/z$. It follows that, since $\mathcal{G}$ is continuous, it reaches a maximum on the interval $\left[ r^{0},r^{s-1} \right]$. Suppose that a maximum is located between two boundaries, say $r^{j}$ and $r^{j+1}$. Then, because$\mathcal{G}$ is linear on the segment $\left[ r^{j},r^{j+1} \right]$, it would necessarily be maximum on the whole segment. Consequently, it suffices to look for maxima of $\mathcal{G}$ among the boundaries $r^{0},\ldots,r^{s-1}$. Using [S12], we can write the expression of $\mathcal{G(}r^{i})$:

|  | $\mathcal{G(}r^{i})=\left( 1-p \right)\left[ \left( 1-p- \frac{1}{z} \right)\frac{s}{s-i}+\sum_{k=0}^{i} \left( \begin{matrix} s \\ k \end{matrix} \right)p^{k}\left( 1-p \right)^{s-k}\frac{k-i}{s-i} \right].$ | [A14] |
| --- | --- | --- |

We now demonstrate that the set $\mathcal{\{G}\left( r^{i} \right)\}$ has a unique maximum. When $s=1$, the set has only one value; $\mathcal{G}$ is therefore maximum for $r^{0}=0$. When $s>1$, we study the sign of the difference $\mathcal{G(}r^{i+1}\mathcal{)-G(}r^{i})$ using Eq. [S14] for $i=0, \ldots, s-2$:

|  | $\mathcal{G}\left( r^{i+1} \right)\mathcal{-G}\left( r^{i} \right)=\frac{s \left( 1-p \right)}{\left( s-i-1 \right)\left( s-i \right)} \left( \theta_{i}\left( p \right)- \frac{1}{z} \right),$ | [A15] |
| --- | --- | --- |

where $\theta_{i}\left( p \right)$ is the term:

|  | $\theta_{i}\left( p \right)=1-p-\sum_{k=0}^{i} \left( \begin{matrix} s \\ k \end{matrix} \right)p^{k}\left( 1-p \right)^{s-k}\left( 1- \frac{k}{s} \right).$ | [A16] |
| --- | --- | --- |

From Eq. [S15], we observe that, unless $p=1$, for which $\mathcal{G}$ is always null, the sign of $\mathcal{G(}r^{i+1}\mathcal{)-G(}r^{i})$ is determined by the relative value of $\theta_{i}\left( p \right)$ and $1/z$. From Eq. [S16], the $\theta_{i}\left( p \right)$s are decreasing when $i$ increases from $0$ to $s-2$. We are left with three possibilities.

1. When $\theta_{0}\left( p \right)<1/z$, we have $\mathcal{G}\left( r^{i+1} \right)\mathcal{<G(}r^{i})$ for $i=0, \ldots, s-2$. This implies that $\mathcal{G}$ is maximum at $r^{0}=0$.
2. When $\theta_{s-2}\left( p \right)>1/z$, we have $\mathcal{G}\left( r^{i+1} \right)\mathcal{>G(}r^{i})$ for $i=0, \ldots, s-2$. This implies that $\mathcal{G}$ is maximum at $r^{s-1}=s-1$.
3. Otherwise, we can find an integer $i^{*}$ between $0$ and $s-2$ such that $\theta_{i}\left( p \right)>1/z$ for $i<i^{*}$ and $\theta_{i}\left( p \right)\leq1/z$ for $i\geq i^{*}$. This implies that $\mathcal{G}$ is maximum at $r^{i^{*}}={i^{*}}/{(s-i^{*}})$.

For particular values of $p$ and $z$, defined by the following equations:

|  | $z=1/{\theta_{i}(p)},$ | [A17] |
| --- | --- | --- |

with $i=0,\ldots, s-2$, we have $\mathcal{G}\left( r^{i+1} \right)\mathcal{=G}\left( r^{i} \right)$. This implies that $\mathcal{G}$ is maximum both at $r^{i}$ and $r^{i+1}$, and therefore on the whole segment $\left[ r^{i}, r^{i+1} \right]$. In these particular cases, we suppose that firms select the smallest overordering rate that maximizes $\mathcal{G}$, i.e., $r^{i}$. In conclusion we have established that, for a combination of $z$ and $p$ values, the profit-maximizing overordering rate is unique. As $z$ and $p$ vary, it can take only a limited set of values: $i/\left( s-i \right)$ with $i=0,\ldots,s-1$. Equation [S17] defines the regions of the $(z,p)$ parameter plane corresponding to the different profit-maximizing overordering rates. These are shown in Fig S3. A small modification of $z$ or $p$ either has no impact on overordering decisions or leads to a discontinuous adjustment. With more suppliers, the number of potentially optimal overordering rates increases; see the growing number of regions in Fig. 3 from panel (a) to (c). Consequently, discontinuities fade out as the number of suppliers grows; see Fig. S4(a). In other words, the wider the supplier base, the more finely firms can adapt to a particular level of risk. The loss due to supplier failures thus decreases as $1/\sqrt{s}$; see Fig. S4(b).


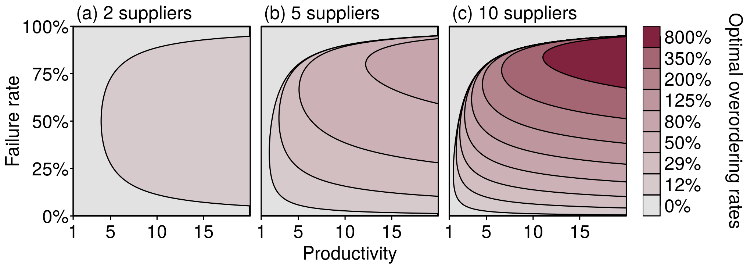


**Fig. S3.** The optimal overordering of fully perishable inputs discontinuously changes with productivity and failure rate. The three panels show, in the parameter plane $(z,p)$, the overordering rates that maximize the profit of a firm supplied by (a) $s=2$, (b) $s=5$ or (c) $s=10$ primary producers. The regions are separated by the curves defined in Eq. [S17].

In the full integration scenario, all firms belong to the same group. They aim to maximize the expected profit of the group, denoted by $\mathbb{E}\left( \pi_{G} \right)$. We infer from the symmetry of the supply chain that all primary producers choose the same overordering rate $r_{P}$ and have the same expected profit $\mathbb{E}\left( \pi_{P} \right)$:

|  | $\mathbb{E}\left( \pi_{P} \right)=\frac{1+r_{F}}{s z}\left( 1-p-\frac{1+r_{P}}{z} \right),$ | [A18] |
| --- | --- | --- |

where $r_{F}$ the overordering rate of the final producer. The group profit $\mathbb{E}\left( \pi_{G} \right)$ is thus equal to $\mathbb{E}\left( \pi_{F} \right)+s\mathbb{E}\left( \pi_{P} \right)$, where $\mathbb{E}\left( \pi_{F} \right)$ is the expected profit of the final producer defined in Eq. [S10]. Since $r_{P}$ only influences $\mathbb{E}\left( \pi_{P} \right)$ and not $\mathbb{E}\left( \pi_{F} \right)$, primary producers choose the overordering rate that maximizes their own profit. From Eq. [S18], this overordering rate is $0\%$, whatever the choice of the final producer. As for the latter, we define on $\mathbb{R}^{+}$ the function $\mathcal{H:}r_{F}\mathbb{⟼E}\left( \pi_{G}(r_{F}) \right)$, which is, as $\mathcal{G}$, piecewise linear and has the same boundaries $r^{i}$ between linear pieces, given in Eq. [S13]. The last linear piece on $\left[ r^{s-1}, +\infty\right]$ is decreasing in $r_{F}$ with the coefficient of variation $-1/{z^{2}}$, which ensures that, as $\mathcal{G}$, $\mathcal{H}$ has a unique maximum among the values $r^{i}$, with $i=0,\ldots,s-1$.


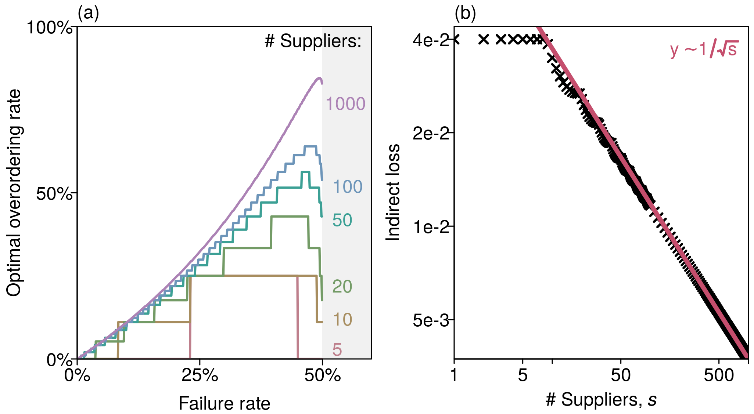


**Fig. S4.** A larger supplier base enables finer adaptation to risks. Both panels concern a single firm supplied by multiple primary producers. Panel (a) shows how the relationship between profit-maximizing overordering rate and failure rate changes with the number of suppliers. In particular, the 5- and 10-supplier curves are the vertical intersects of Fig. S3(b) and (c) for $z=2$. Note that over 50% failure rate, in the grey region, firms are unproductive. Panel (b) presents how the expected indirect loss decreases with the number of suppliers for a failure rate of 10%. The red curve is a fit, showing that indirect loss decreases as $1/\sqrt{s}$.

## S5. Algorithmic computation of the expected profits in fully connected and layered networks

In large acyclic networks, we cannot write an explicit expression of expected profits for each firm. For the general case, we approach these values through the numerical simulations of the stochastic process. For a specific class of structure, however, we can write a deterministic algorithm that allows us to access the exact values of the expected profit. An example of such a network is shown in Fig. 1(b). It is said to be “layered,” because firms are organized in layers: at layer 1 lie the primary producers, at layer 2 the direct customers of the primary producer, and at layer 3 the direct customers of layer-2 firms, and so on all the way to final producers. It is said to be “fully connected,” because each firm at one layer is supplied by all the firms of the layer underneath, except primary producers which are externally supplied. The topology is described by the number of firms $n^{l}$ occupying each layer $l=1, \ldots, L$, where $L$ denotes the number of layers. In this class of supply chain, all firms occupying the same layer share the exact same suppliers and customers. They all face the exact same demand and the same supply risk. This symmetry allows us to make the following analytical simplification: all firms at one layer have the exact same behavior. In particular, they choose the same overordering rate $r^{l}$. The production targets of each firm can either be computed using the input–output relationships, as proposed in the Main Text for general acyclic networks, or using the following relationships:

|  | $\left\{ \begin{aligned} \bar{y^{l}}=\frac{1}{s^{l}} \text{for} l=L, \\ \bar{y^{l-1}}=\frac{s^{l}\bar{y^{l}}\left( 1+r^{l} \right)}{s^{l-1}z} \text{for} l=1, \ldots, L-1. \end{aligned} \right.$ | [A19] |
| --- | --- | --- |

In addition, all firms at one layer simultaneously receive the exact same amount of inputs. We denote by $Q^{l}$ the random variable representing the quantity of inputs received by a layer-$l$ firm. The random variable $Q^{l}$ is discrete; we denote by $q_{j}^{l}$ each of its $j_{l}$ realizations, with $j=1, \ldots, j_{l}$. The core of the algorithm consists of computing, layer by layer, the probability distribution of $Q^{l}$. After computing all production targets $\bar{y^{l}}$ using Eq. [S19], we initialize the process with the primary producers, $l=1$. The probability distribution of $Q^{1}$ is $\bar{y^{1}}/z\left( 1+r^{1} \right)$ with probability one. Next, suppose that we know the probability distribution of $Q^{l}$ (i.e., the value of its realization $q_{j}^{l}$ and its associated probability $\Pr\left( Q^{l}=q_{j}^{l} \right)$). To find the probability distribution of $Q^{l+1}$, we distinguish between $n^{l}+1$ cases, with each case corresponding to the number of direct layer-$l$ firms perturbed: case (1) no firm is perturbed; case (2) one firm is perturbed; case (3) two firms; and so on up to case $\left( n^{l}+1 \right)$ in which all of the $n^{l}$ firms are perturbed. For each case, we do the following computation.

1. We evaluate the probability of the case using combinatory formulas. For instance, the probability of case $(i+1)$, in which $i$ layer-$l$ firms are perturbed, is $\binom{n^{l}}{i}p^{i}\left( 1-p \right)^{l-i}$.
2. We determine the probability distribution of the total quantity produced by layer-$l$ firms, using the known probability distribution of $Q^{l}$ and production targets $\bar{y^{l}}$. For the case $i+1$, the total quantity produced by layer-$l$ firms is $\left( n^{l}-i \right)\min\left( zQ^{l},\overline{y^{l}} \right)$.
3. We deduce the probability distribution of inputs received by layer-$\left( l+1 \right)$ firms. For the case $i+1$, each layer-$\left( l+1 \right)$ firm receives $\left( n^{l}-i \right)/{n^{l+1}}\min\left( zQ^{l},\overline{y^{l}} \right)$ inputs.

Each case thus provides a part of the probability distribution of $Q^{l+1}$. Case $\left( i+1 \right)$ provides the following potential realizations of $Q^{l+1}:$

|  | $\frac{\left( n^{l}-i \right)}{n^{l+1}}\min\left( zq_{j}^{l},\overline{y^{l}} \right), \text{for} j=1, \ldots,j_{l},$ | [A20] |
| --- | --- | --- |

along with their corresponding probabilities:

|  | $\binom{n^{l}}{i}p^{i}\left( 1-p \right)^{n^{l}-i}\Pr\left( Q^{l}=q_{j}^{l} \right), \text{for} j=1, \ldots,j_{l}.$ | [A21] |
| --- | --- | --- |

We recombine all of the $n^{l}+1$ cases to elicit the complete probability distribution of $Q^{l+1}$. By applying the recursive process from primary producers all the way to final producers, we can determine the probability distributions of $Q^{l}$ for all $l=1, \ldots, L$. Next, we deduce directly from these distributions the probability distribution of the sales, denoted by $Y^{l}$. Sales are null with probability $p$ or otherwise equal to $\min\left( zQ^{l},\overline{y^{l}} \right)$. Last, knowing the probability distribution of the inputs and sales of each firms, we can compute the expected profits $\mathbb{E}\left( \pi^{l} \right)\mathbb{=E}\left( Y^{l}-Q^{l} \right)$.

## S6. Detailed description of the evolutionary process

The optimization problem of each firm is to find the overordering rate that maximizes the profit of the group it belongs to. We define an evolutionary process to elicit the solutions. We call strategy the choice of a particular overordering rate. The set of strategies for which no firm has any interest in choosing another strategy is called the set of evolutionarily stable strategies (ESSs); it corresponds to a Nash equilibrium. We call fitness landscape of firm $i$ at evolutionary step $\tau$ the mapping of its overordering rate $r_{i}^{\tau}$ into the expected profit of the group $\mathbb{E(}\pi_{g}^{\tau} )$; this is determined by the topology $M$, by the firm-level parameters$z$, $v$ and $p$, and by the overordering rates of the other firms $r_{j\neq i}^{\tau}$. The evolutionary process proceeds through gradient ascent. One firm at a time performs an explore-and-adjust procedure: it explores its fitness landscape by testing different strategies, then makes a small adjustment to move up the landscape. We denote by $\mathcal{U}_{\tau}$ the ensemble of firms that have not reached their ESS at the evolutionary step $\tau$. The initial ensemble $\mathcal{U}_{0}$ gathers all firms except primary producers. Since the latter firms receive riskless supply of raw materials, they always find it more profitable not to overorder. An initial overordering rate of typically $0\%$ is attributed to each firm. At evolutionary step $\tau$, firms in $\mathcal{U}_{\tau}$ perform the explore-and-adjust procedure, one after the other, in a random order. Exploration consists of three trials, under which three strategies are tested: $max(0,r_{i}^{\tau}-\Delta), r_{i}^{\tau}$ and $r_{i}^{\tau}+\Delta$. To test a strategy, we run the model over $T$ time steps and compute the corresponding time-averaged group profit $\left\langle\pi_{g}^{\tau} \right\rangle_{T}$. For the three tests, we apply the same sequence of perturbations, randomly generated by $n$ simultaneous Bernoulli processes—one per firm—of length $T$ and probability $p$. Using the same sequences of perturbations corresponds to the variance reduction technique called common random numbers. The three pairs $\left\{ r_{i}^{\tau},\left\langle\pi_{g}^{\tau} \right\rangle_{T} \right\}$ are used to estimate the local geometry of the fitness landscape. An estimate of the gradient, denoted by $\gamma_{i}^{\tau}$, is given by the derivative in $r_{i}^{\tau}$ of a parabola fitted in a least square sense to the three points. The overordering rate of firm $i$ is then updated as follows: $r_{i}^{\tau}=r_{i}^{\tau}+B_{\delta}(h \gamma_{i}^{\tau})$, where $h$ is a scaling constant and $B_{\delta}$ a bounding function that returns $\delta$ if its argument exceeds $\delta$, -$\delta$ if its argument falls below $-\delta$, or the argument itself otherwise. These bounds limit the size of the updating step. Once all firms in $\mathcal{U}_{\tau}$ have gone through this procedure, all updated strategies are carried forward: $r_{i}^{\tau+1}=r_{i}^{\tau}$. Some firms may have reached their ESS and will therefore be removed from the new ensemble of updating firm $\mathcal{U}_{\tau+1}$. The whole process is repeated until $\mathcal{U}_{\tau}$ becomes empty. A firm is considered to have reached its ESS if its strategy has become stationary. Stationarity is tested as follows. Within an evolutionary-time window of length $W$, which spans the interval $\left[ \tau-W+1, \tau\right]$ , we randomly select two smaller windows that are longer than $W/2$. An evolutionary time series is considered stationary when the linear trend on each one of the two windows falls below a threshold $w$. In this case, the ESS is estimated by the average value over the last $W$ steps. Table S1 summarizes the relevant parameters and their values. The stochastic nature of the external perturbations poses several challenges to the design of the evolutionary process. The three trials only provide estimates of the fitness landscape. When each trial is made under different sequences of perturbations, we observe that the estimated fitness landscape sometimes leads to ambiguous results. Specifically, as soon as the local geometry of the expected fitness landscape becomes flatter, the estimated landscape becomes rugged, even with longer duration of the trials, such as $T=10,000$. Instead, when the three trials use a common sequence of perturbations, they produce comparable results and enable unambiguous adjustments. This solution, however, generates evolutionary fluctuations. Since a new sequence of perturbations is used at each evolutionary time step, the estimation of the same fitness landscape may slightly vary. These varying estimates produce noises in the evolutionary time series of the strategies, as observed in Fig. 1(a) of the main text. Consequently, to determine whether a firm has reached its ESS, we use a stationarity condition. Note that, using the same sequence of perturbation over a whole simulation would lead to overordering decisions that are adapted to this precise sequence but not to the overall failure rate, leading to large variability across simulations. In Tab. S1, we selected the parameter values that lead, within reasonable computation time, to reproducible results: the standard deviations over the evolutionary stable overordering rates is below $1\%$. For fully connected and layered networks, illustrated in Fig. 1(b), we can use the algorithm of Sec. S5 to obtain the exact expected profits. At each evolutionary time step, we have access to the exact fitness landscape. For this class of supply chain, we can therefore determine the exact ESS.

In the baseline scenario—a fully fragmented supply chain with $n=30$ and $c=2$, we report the following computational metric. On a laptop with an Intel i7-7500U CPU @ 2.70 GHz, the evolutionary equilibrium is reached on average after 70 seconds. CPU usage reaches 35% and memory usage is about 68 Mo. We observe that the computation time scales as $nln(n)$.

**Table S1.** Summary of the parameters used in the evolutionary process

| Parameter | Definition | Value |
| --- | --- | --- |
| $T$ | Duration of the trials, in economic time step | $100$ |
| $\Delta$ | Exploration step | $5.{10}^{-2}$ |
| $\delta$ | Maximum updating step size | $2.{10}^{-2}$ |
| $W$ | Evolutionary time window for testing stationarity | 30 |
| $w$ | Stationary threshold | ${10}^{-4}$ |

## S7. Evidences of the existence and uniqueness of Nash equilibria

As studied in Secs. S2 to S4, a firm supplied by multiple primary producers has a unique solution to its optimization problem, both in the fully fragmented and in the fully integrated scenarios. For specific combinations of $z$, $v$ and $p$ — e.g., Eq. [S17] — a continuous range of strategies could solve the optimization problem. To ensure uniqueness, we have assumed that firms have a waste-minimizing behavior: when a range of overordering rates yield similar outcome, they choose the smallest one. The uniqueness of the solution applies to any two-layer supply chains. Strategic interactions occur in a supply chain of three or more layers. The strategy chosen by a layer-2 firm influences the fitness landscape of layer-3 firms. In fact, extensive numerical simulations show that all fitness landscapes have a unique maximum, as studied in Sec. S4. Examples are shown in Fig. S5 for $v=0\%$, both for the fully fragmented scenario in panels (b) and (e) and for the fully integrated one in panels (c) and (f). In fully connected and layered network, as in Figs. S5(a–c), the number of linear pieces increases as we move down the supply chains. In a random acyclic network, as in Figs. S5(d–f), the fitness landscape become even smoother. This property of the fitness landscapes suggests that a unique Nash equilibrium exists generically in a supply chain. Numerical experiments using a very diverse set of initial conditions show that the evolutionary process leads to similar ESSs, within $1\%$ standard deviation. The remaining variability is attributed to the fluctuations discussed in Sec. S6.


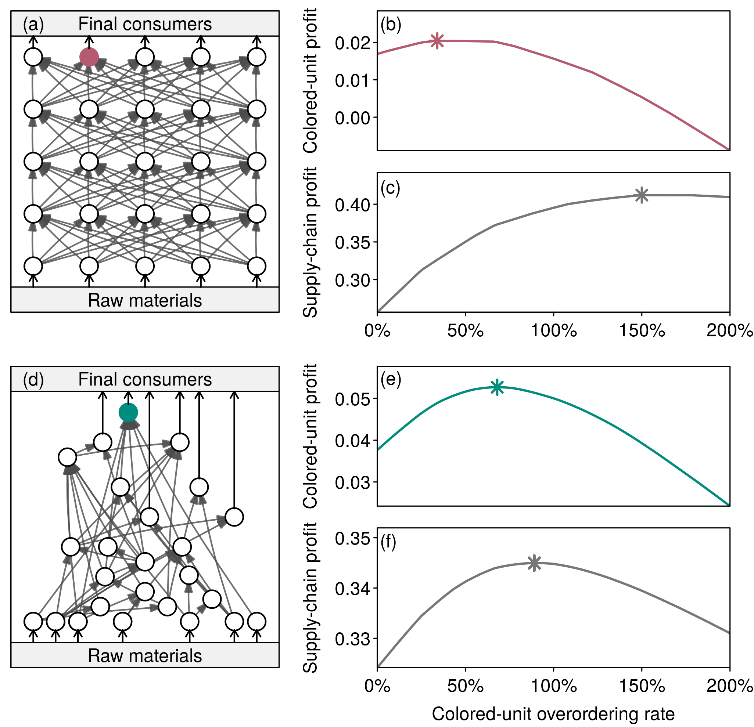


**Fig. S5.** Fitness landscapes always have a unique maximum. Panels (b–c) (resp. panels (e–f)) show the fitness landscape at ESS of the colored firm of the supply chain displayed in panel (a) (resp. panel (d)). Panels (b) and (e) concern full fragmentation: the fitness landscape is the relationship between the colored-firm profit and its overordering rate. Panels (c) and (f) concern full integration: it shows the relationship between the profit of the whole supply chain and the colored-unit overordering rate. In all panels, $p=30\%$, $z=2$. Durability is $0\%$ in panels (b–c) and $50\%$ in panels (e–f).

## S8. Periodic patterns of inventory emerging from threshold behavior

As mentioned in the main text, two main mechanisms shape the overordering decisions. First, each supplier, both direct and indirect, is a potential source of hazard, leading to stronger overordering for firms located downstream. Next, the inventory maintained by a supplier may sufficiently dampen the disruption cascade, so that its clients may overorder less. In a random acyclic network, the first mechanism dominates, as shown in Fig. 1(c) of the main text, but the second generates a large variability. For layered structures, however, firms belonging to the same layer share a very similar position and tend to act alike. Consequently, the second mechanism becomes more detrimental in shaping the outcome. Figure S6 presents the periodic pattern that emerges from these interactions in a fully connected and layered network. According Sec. S4, with two suppliers and non-durable goods, the overordering rates that maximize the profit of firms is either $0\%$ or $100\%$. Clients of primary producers face risks that are not high enough, so that overordering is not profitable. As we move down, risks pile up. At layer 9 a threshold is crossed and a $100\%$-overordering becomes profitable. Because of this decision, the level of risks is reduced so much that layer-10 firms have no incentive to overorder. As we move the next layers, risks pile up again until a new threshold is met, and overordering becomes profitable, at layer 15. This sequence repeats itself and leads to a periodic pattern of length 5.


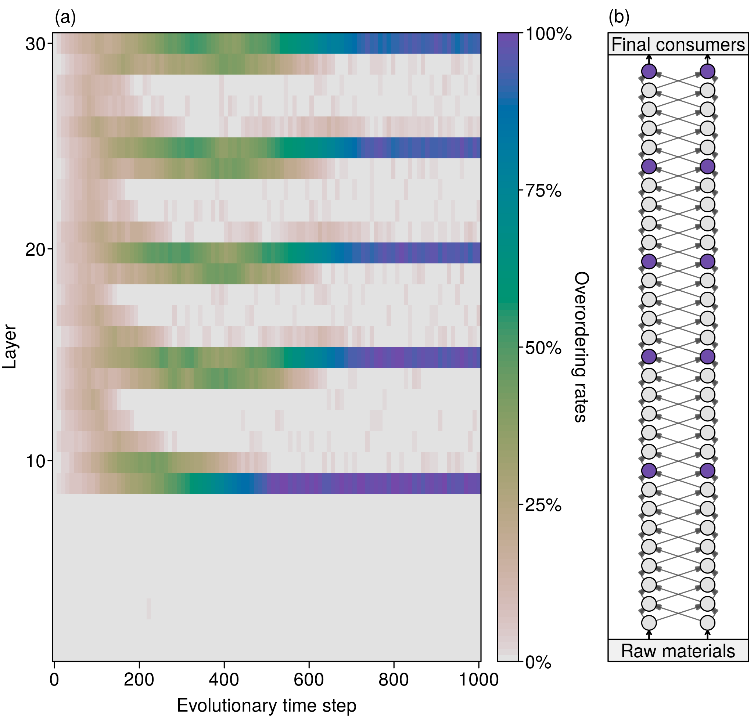


**Fig. S6.** A periodic overordering pattern emerges from strategic interactions. Panel (a) presents the evolutionary dynamics corresponding to the full fragmentation scenario that settles in the fully connected 30-layer supply chain with 2 firms per layer displayed on panel (b). The average overordering rate of each of the 30 layers is indicated by a color code: grey corresponding to no overordering and purple to a 100%-overordering rate. Panel (b) displays the resulting pattern: only firms located at layers 9, 15, 20, 25, and 30 overorder.

## S9. Robustness check of the negative impact of fragmentation on mitigation

Figure 3 of the main text shows how the average mitigation success changes with fragmentation for six classes of acyclic random network. Each point of these curves is, for a given level of fragmentation, an average over 20 supply chains, and for each supply chain with $n$ firms, over $10\times n$ group configurations. Using the same colors, each panel of Fig. S7 presents the dispersion of underlying data. The top subpanels show the inter-network dispersion: how mitigation success, averaged over the group configurations, varies across the 20 networks. The bottom subpanels present the inter-group dispersion: how mitigation success, scaled by the average over all networks, varies across the $10\times n$ group configurations. Such variability is sizeable, indicating that, for specific group configurations, a slightly more fragmented supply chain may have a larger mitigation success. But the overall finding remains robust: in average fragmentation hampers risk mitigation. The variability between networks is much larger, indicating that the overall mitigation success is strongly influenced by the structure of the supply chain. This finding points toward further investigations aiming at identifying the structural features that influence risk mitigation.


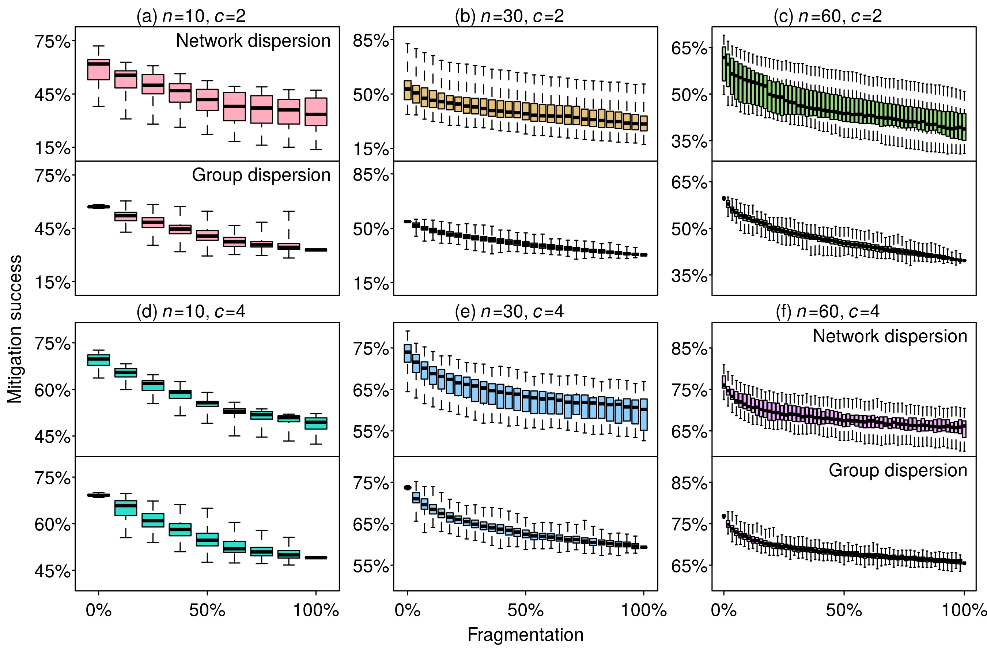


**Fig. S7.** Dispersion of the data underpinning the six curves of Fig. 3 of the main text. Each panel corresponds to one of the curves. For each fragmentation level, two distributions of mitigation success are shown. The top one represents the network dispersion: it is made up of 20 data points, which are the average mitigation success over the $10\times n$ group configuration generated for each network. The bottom one represents the group dispersion, made of $20\times10\times n$ data points. To show only the group-configuration-induced variability, we remove the network dispersion effect by rescaling each data point by the average mitigation success. Specifically, each data point is the mitigation success obtained with one network and one group configuration, minus the average mitigation success over all configurations for the specific network, plus the average over all configurations and all networks. The bottom and top of the boxes indicate the first and third quartiles, the heavy horizontal lines inside the boxes pinpoint the medians, and the whiskers extend to the extrema.

## S10. Predicting the optimal overordering rates with network indicator

Could the optimal overordering rate of a particular firm be predicted by its position in the supply chain, without an explicit analysis of the strategic interactions? We use linear and multilinear regression analyses to test the ten network indicators presented in Table S2. Each of these captures a particular aspect of the position of the firm in the supply chain. Three indicators focus on the supply side: the number of direct suppliers, the number of total suppliers, and the supply-chain level. Three others are their counterparts for the demand side: the number of direct clients, the number of total clients, and the inverted supply-chain level. The number of similar firms captures the density of firms sharing the same clients. The last three indicators are centrality measures: closeness and betweenness centrality, page ranks.

**Table S2.** Summary of the ten network indicators.

| Name | Explanation |
| --- | --- |
| # Direct suppliers | Number of direct suppliers. |
| # Total suppliers | Number of all direct and indirect suppliers, up to primary producers. |
| Supply-chain level | Average length—i.e., number of links—of all pathways connecting the firm to primary producers. This is equivalent to the ecological trophic level (1). |
| # Direct clients | Number of direct clients. |
| # Total clients | Number of all direct and indirect clients, up to primary producers. |
| Inverted supply-chain level | Average length of all pathways connecting the firm to final producers. This is the supply-chain level of the network in which all links have been reverted. |
| # Similar firms | Number of other suppliers of the firm’s clients |
| Closeness centrality | Inverse of the sum of the length of the shortest path connecting the firm to all others (2). |
| Betweenness centrality | Importance in connecting any two regions of the network (2).^32^(Newman 2010) |
| Page ranks | Importance in supplying clients that are themselves important suppliers (2). |

We performed linear and multilinear regressions of the indicators on the ESSs, both full fragmentation and full integration. We exhaustively tested combinations of indicators, using stepwise algorithms for model selection. For each scenario, the dataset contains 60,000 points, corresponding to 2,000 directed acyclic random networks with $n=30$, $c=3$, $z=2$, $v=50\%$, and $p=10\%$. We first performed linear regressions using one indicator at a time. Results are presented in Table S3. The highest coefficient of determination is reached by the number of total suppliers in the fragmentation scenario and by the supply-chain level in the integration scenario. Overall, the indicators focusing on the supply side, including page rank, outperform those focusing on the demand side. This difference is slightly reduced in the integration scenario, in which the impact of overordering on clients is taken into account by firms. We note, in particular, that closeness and betweenness centrality do not capture relevant information for the allocation of overordering rates. These results confirm the findings shown in Fig. 5 of the main text. The overordering rates leading to the higher mitigation success are those of the full integration scenario. Therefore, supply-chain level is the most accurate tool that decision-makers can use to find an allocation of overordering rates that reduces systemic risks. With full fragmentation, after removing the variability captured by the number of total suppliers, a large dispersion of the overordering rate remains, as seen in Fig. 1(c) of the Main Text. No other indicator, however, is able to capture the remaining variability. Even a multilinear model containing all indicators increases the coefficient of determination by only 0.03. With full integration, a similar multilinear model increases the coefficient of determination more significantly, from 0.48 with supply-chain level only to 0.62. Using stepwise model selection, we found that the inverted supply-chain level is the second best indicator. This result confirms that, in integrated supply chains, the position of a firm in relation to its clients could also be taken into account to improve the allocation of overordering rates. The large remaining variability, however, suggests that strategic interactions fail to be captured by linear models. Decentralized decisions always outperform centralized ones based on linear models.

**Table S3**. Results of the linear regressions made for each indicator of Table S2 on the evolutionary stable overordering rates of individual firms for the fully fragmented and fully integrated scenarios. Each regression contains only one indicator at a time. For each scenario, the indicator with the highest coefficient of determination is highlighted. All p-values are below ${10}^{-10}$. Each dataset contains 60,000 points, corresponding to 2,000 networks with parameters $n=30$, $c=3$, $z=2$, $v=50\%$ and $p=10\%$.

| Indicator | Full fragmentation | | Full integration | |  |
| --- | --- | --- | --- | --- | --- |
|  | Estimated coefficient  (standard error) | Coefficient of determination | Estimated coefficient  (standard error) | Coefficient of determination | |
| # Direct suppliers | 0.033  (0.00018) | 0.59 | 0.052  (0.00060) | 0.20 | |
| # Total suppliers | **0.016**  **(0.000049)** | **0.69** | 0.023  (0.00017) | 0.40 | |
| Supply-chain level | 0.042  (0.00027) | 0.49 | **0.11**  **(0.00066)** | **0.48** | |
| # Direct clients | -0.019  (0.00024) | 0.20 | -0.060  (0.00058) | 0.26 | |
| # Total clients | -0.0064  (0.000079) | 0.21 | -0.018  (0.00019) | 0.24 | |
| Inverted supply-chain level | -0.030  (0.00033) | 0.26 | -0.096  (0.00073) | 0.36 | |
| # Similar firms | -0.0054  (0.000095) | 0.12 | -0.019  (0.00022) | 0.19 | |
| Closeness centrality | 9.97  (0.21) | 0.084 | -3.41  (0.54) | 0.0013 | |
| Betweenness centrality | 0.0014  (0.000069) | 0.017 | -0.0022  (0.00017) | 0.0055 | |
| Page ranks | 2.34  (0.014) | 0.55 | 4.42  (0.042) | 0.27 | |
